# Patient With MGRS/PGNMID Without Detection of a Peripheral Clone: Case Report and Literature Review

**DOI:** 10.1155/crh/8043845

**Published:** 2026-05-10

**Authors:** Agatha Larrazábal, Ana Ávila-Rodriguez, Matías Sánchez

**Affiliations:** ^1^ Department of Hematology at Fundación Arturo López Pérez, Santiago de Chile, Chile; ^2^ Facultad de Medicina, Subespecialidad de Hematología, Universidad de los Andes, Santiago de Chile, Chile, uandes.cl; ^3^ Department of Medicine, Division Hematology/Oncology University of Illinois Chicago-UI Health, Chicago, Illinois, USA

**Keywords:** acute kidney injury (AKI), clone-negative PGNMID, daratumumab, monoclonal gammopathy of renal significance (MGRS), nephrotic syndrome, proliferative glomerulonephritis with monoclonal immunoglobulin deposits (PGNMID)

## Abstract

Monoclonal gammopathy of renal significance (MGRS) is a clonal cell proliferative disorder, characterized by the production of monoclonal immunoglobulins in patients that do not meet hematological criteria for a specific malignancy. It can be present in B cell and plasma cell clonal proliferative diseases and accounts for 10% of monoclonal gammopathy of undetermined significance (MGUS) cases. We present a case of a patient presenting with acute kidney injury, hematuria, and nephrotic syndrome, who after renal biopsy was diagnosed with a proliferative glomerulonephritis with monoclonal immunoglobulin deposits (PGNMIDs), a subtype of MGRS, and treated with chemoimmunotherapy (daratumumab, cyclophosphamide, bortezomib, and dexamethasone), with complete renal function recovery.

## 1. Introduction

The International Kidney and Monoclonal Gammopathy Research Group established monoclonal gammapathy of renal significance (MGRS) as a term to identify a group of clonal plasma cell proliferative disorders that produce nephrotoxic monoclonal immunoglobulins without meeting diagnostic criteria of malignancies such as B cell and plasma cell clonal proliferative disorders [1]. MGRS accounts for about 10% of monoclonal gammopathy of undetermined significance (MGUS) cases, affecting approximately 0.32% of individuals older than 50% and 0.53% of those older than 70 [2], and unlike patients with MGUS, these patients’ clonal disorders cause kidney damage which eventually trigger their diagnostic studies.

MGRS can be classified into 7 different subtypes, according to the deposits found in these patients’ kidneys: monoclonal immunoglobulin deposition diseases (including light chain, heavy chain, and light/heavy chain deposition disease), amyloidosis related (most commonly light chain amyloidosis), proliferative glomerulonephritis with monoclonal immunoglobulin deposits (PGNMIDs), immunotactoid and fibrillary glomerulonephritis, cryoglobulinemic glomerulonephritis (type 1), C3 glomerulopathy with monoclonal gammopathy, and tubulointerstitial nephropathies. Even though they often share certain clinical features, they ultimately have different prognosis and outcomes.

PGNMIDs are characterized by the deposition of monoclonal immunoglobulins within the glomeruli (especially IgG); however, nephropathic clones are identified only 30% of the time. The pathogenesis still remains unclear, as it can occur in any age group and in many different clinical settings, like malignancy, infection, or in the context of MGRS [3]. They account from 0.17% to 3.7% of renal biopsies [4] and occur more frequently in 50‐year‐old patients. The clinical manifestations are nonspecific; patients may present with renal insufficiency, hypertension, hematuria, proteinuria, and nephrotic syndrome.

We present a case report on a 61‐year‐old female with no previous known medical history of hematologic or renal disorders, who presents with rapid onset nephrotic syndrome and acute kidney injury (AKI). Among diagnostic studies, she had a creatinine of 6.5 mg/dL, hematuria, and 10 g of albuminuria; normal serum immunofixation electrophoresis (SIFE) and urine immunofixation electrophoresis (UIFE); normal ratio of serum kappa/lambda free light chains (FLCs); and normal bone marrow (BM) biopsy with the absence of plasma cell dyscratias. Renal biopsy supported the diagnosis of PGNMID/MGRS. Initially treated with high‐dose steroids, renin angiotensin aldosterone (RAAS) inhibitors, and renal replacement therapy, with partial response, once confirmed with PGNMID diagnosis, she was treated with chemoinmunotherapy: daratumumab, cyclophosphamide, and dexamethasone (daratumumab CyborD) recovering with an excellent renal function.

This case exemplifies the difficulties that physicians face when diagnosing MGRS‐related renal disorders, as in some instances, there will be an absence of serum clonality detection regardless of the damage being produced in the renal compartment. We intend to discuss the rarity of this disease, the available information regarding diagnosis, specifically under what circumstances are renal biopsies necessary to support diagnostic suspicions and what is the best available treatment for these patients.

## 2. Case Presentation

A 61‐year‐old female with a past medical history of neurocognitive disorder, major depressive disorder with psychotic features, hyperlipidemia, gastritis, osteopenia, well‐controlled hypertension, and active smoking, who consults in the nephrology clinic referring 2 weeks of increased generalized grade 2+ pitting edema with progressive dyspnea of exertion compatible with NYHA IV, without known medical history of heart failure or respiratory issues. Physical examination with notorious generalized anasarca, bilateral leg edema grade 2+ pitting up to knee, and bilateral basal pulmonary rales.

Exams on admission are shown in Table 1.

**TABLE 1:** Exams on admission.

Test	Result	Range
Hemoglobin (g/dL)	9.0	13.5–17.5
Mean corpuscular volume (MCV) (fL)	85.3	80–100
MCHC (mean corpuscular hemoglobin concentration) (g/dL)	33.7	320–36
WBC (10^3^/μL)	9.8	4.5–11.0
Neutrophils (%)	83.5	40–60
Lymphocytes (%)	8.9	20–40
Creatinine (mg/dL)	6.59	0.7–1.1
Blood urea nitrogen (BUN) (mg/dL)	63	7–20
Sodium (mEq/L)	126	135–145
Potassium (mEq/L)	4.0	3.5–5.1
Chloride (mEq/L)	84	98–107
Albumin (g/dL)	2.6	3.5–5.0
Calcium (mg/dL)	7.3	8.5–10.5
24‐hour urine collection (grams of protein/24 h)	10.3	< 0.15
Hematuria	+++	−

The rest of the exams were unremarkable.

In the study of differential diagnosis for nephrotic/nephritic syndromes, autoimmune related glomerulopathies were ruled out: negative ANA, anti‐GBM, PR3, MPO, anticardiolipin (IgM, IgG, and IgA), antiB2glycoprotein, lupus anti anticoagulant, and normal complement C3 of 96 (Ref: 75–180 mg/dL). Also, negative infectious workup for Hepatitis B and C. Paraprotein studies were requested, with no monoclonal protein detected through SIFE and UIFE, and serum kappa/lambda light chains in a normal ratio of 1.68 (Ref: 0.26–1.65). While slightly above the standard reference range, this value was well within the validated renal reference range (0.37–3.10) for patients with significant kidney dysfunction, indicating the absence of a detectable monoclonal light chain excess.

**FIGURE 1:**
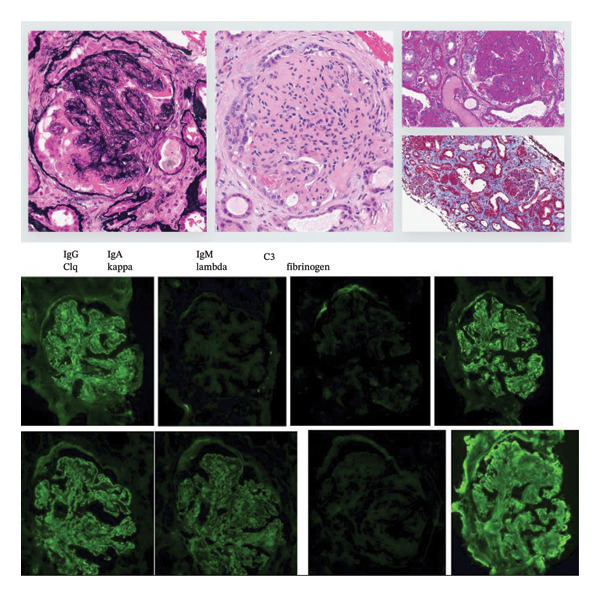
Light microscopy (LM) and immunofluorescence (IF) for IgG subclass staining: MPGN pattern of injury and immunofluorescence (strong diffuse global mainly peripheral C3, C1q, IgG kappa light chains and trace IgM, IgG3 subtype) supporting diagnosis of proliferative glomerulonephritis with monoclonal immunoglobulin deposits (PGNMID)/monoclonal gammapathy of renal significance (MGRS).

Further exams done in the following days to admission are as follows:

• Kidney biopsy: MPGN pattern of injury and immunofluorescence (IF) (strong diffuse global mainly peripheral C3, C1q, IgG kappa light chains and trace IgM, IgG3 subtype) supporting the diagnosis of PGNMIDs/MGRS. See Figure 1 for light microscopy (LM) and IF for IgG subclass staining, which shows strong diffuse global mainly peripheral C3, C1q, IgG kappa light chains and trace IgM, IgG3 subtype.
• BM biopsy: erythroid elements adequate with full spectrum of maturation, no dysplasia; myeloid elements adequate with full spectrum of maturation, no dysplasia or increased blasts; megakaryocytes adequate with normal morphology. Plasma cells not increased and with no atypical features. CD138 IHC shows occasional plasma cells. Kappa ISH and lambda ISH show a polyclonal pattern. Normal BM karyotype and FISH.

Awaiting for renal and BM results, she received pulse steroids: methylprednisolone 250 mg IV for 3 consecutive days followed by prednisone 60 mg PO daily and weaned to 40 mg a day with a plan to progressively lower steroid dose until 5 mg a day. She was also started on oral antiproteinuric medications, like lisinopril 20 mg/day, nifedipine 30 mg/day, carvedilol 12.5 mg BID, bumex 6 mg BID, and metolazone 5 mg daily. She received renal replacement therapy twice mainly for fluid overload, afterward the management of edema with diuretics.

**FIGURE 2:**
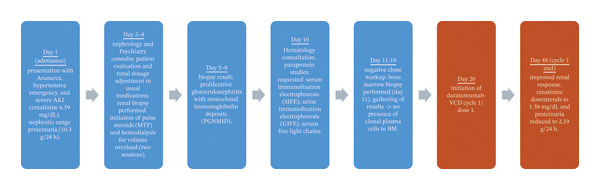
Patient timeline.

After an official diagnosis of PGNMID/MGRS, she was transferred to the hematology unit for further treatment and care. Following the initiation of chemoimmunotherapy with the Daratumumab‐VCD regimen, consisting of subcutaneous daratumumab (1800 mg fixed dose), subcutaneous bortezomib (1.3 mg/m^2^), oral cyclophosphamide (1.5 mg per m^2^), and dexamethasone 40 mg weekly. The patient timeline is further explained in Figure 2.

**FIGURE 3:**
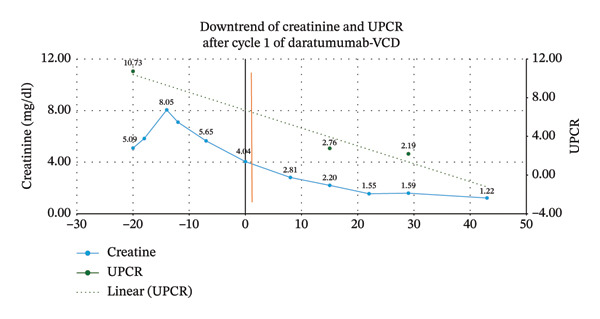
Graphic representation of the downtrend of creatinine (blue line) and proteinuria/creatinuria ratio (UPCR), before and after the initiation of cycle 1 of daratumumab + CyborD chemoimmunotherapy. Vertical yellow line represents the initiation of corticosteroid therapy, and orange vertical line represents Day 0 of initiation of chemotherapy.

The patient showed a rapid biochemical response. As illustrated in Figure 3, serum creatinine decreased from 10.73 mg/dL to 2.19 mg/dL within the first 30 days of treatment.

## 3. Discussion

MGRS is suspected in patients with MGUS and unexplained renal function deterioration and/or proteinuria, frequently progressing to end‐stage renal disease (ESRD) before a timely diagnosis [5]. As previously mentioned, various mechanisms by which monoclonal Igs can cause kidney damage include intratubular precipitation, tissue deposition, fibrillogenesis, crystallization, complement activation, and cytokine activation. In some cases, detection of the pathologic clone or monoclonal protein is not possible, making it difficult to diagnose and treat MGRS.

The diagnosis of MGRS requires a kidney biopsy. The most commonly used classification system is the one introduced by the International Kidney and Monoclonal Gammopathy Research Group (IKMG), which categorizes these lesions based on the IF characteristics and the properties of the deposits observed under electron microscopy (EM).

There can be no discrepancies between the monoclonal gammopathy and the monoclonal protein found in the kidney and, therefore, a renal pathologist should screen for monoclonal protein deposits in all of the compartments of a renal biopsy specimen. Even though the updated classification considers the ultrastructural appearance of the deposits on EM, this is not widely available and, therefore, LM and IF studies with a full panel of antibodies are required [6].

Deciding which patient with acute (AKI) or chronic kidney disease (CKD) and monoclonal gammopathy should undergo a kidney biopsy to diagnose MGRS remains a challenge, and the identifiable factors that would help clinicians have an assertive use of diagnostic tools have been a matter of discussion. Precisely with this concern in mind, the Mayo MGRS Prediction Tool was developed after the review of 280 cases, with the purpose of predicting which patients are at the higher risk of having MGRS lesions and, therefore, would benefit the most from kidney biopsies. This prediction tool includes 8 variables and patients who have presence of proteinuria, altered UPEP, UIFE, and/or altered FLC relation have the highest positive predictive value before the kidney biopsy; on the contrary, patients with diabetes and high C3 complement levels have a lesser probability of finding MGRS in their kidney biopsy [7]. Another retrospective study, also searching for predictable factors among a 160 patient cohort with MGRS diagnosis, divided risk factors in hematologic and nonhematologic: Of the hematologic parameters, patients with higher FLC levels had an increased likelihood of finding MGRS lesions; and on the nonhematologic factors, a urinary protein > 1.5 g/dL, hematuria and lower C3 complement level were associated with increased risk of finding an MGRS lesion [8].

Considering these tools, our patient lacked the most crucial hematologic value for suspicion of plasma cell dyscratias: the presence of an identifiable clone in serum, BM, or urine. Also, the investigation for AKI with nephrotic syndrome’s differential diagnosis was unfruitful, having ruled out most of the associated infectious and autoimmune conditions that might have been responsible for this clinical presentation. Despite the severe renal pathology, repeated hematological screening—including serum immunofixation and BM flow cytometry—failed to identify a measurable monoclonal plasma cell population, and finally, a kidney biopsy was performed and surprisingly enough, the patient was diagnosed with MGRS/PGNMID. In retrospect, by evaluation through the Mayo/MGRS prediction tool, she had a predictive score of 22%, which creates an opportunity to consider that this validation tool may not be ready for prime time, as it does not accurately predict the probability of MGRS in the small population without a detectable blood clone [9].

Resembling our patient’s presentation and opposed to other MGRS renal disorders, PGNMIDs are distinctively associated with the inability to identify a serum nephropathic clone in up to 70% of the cases [10], making this a very hard diagnosis to confirm. Considering the rarity of this disease, it is probably frequently overlooked since renal biopsies and skilled renal pathologists are not always available to clinicians in the time necessary to make an adequate diagnosis.

In relation to therapy, some authors divide MGRS into four groups: conservative therapy, plasma cell clone (PC)–directed therapy, lymphocytic clone (LC))–directed therapy, and the nonclone‐directed therapy. In those with PGNMID without detectable clone in serum, but normal renal function and no proteinuria, the suggested treatment is conservative therapy (RAAS system inhibitors) for 3–6 months, and depending on response, continue this same line of treatment or switch to clone directed therapy. On the other hand, in those with unstable renal function, the appropriate treatment would be clone directed therapy. In the cases of MGRS; if it is clonal IgM, treatment would be for LC and on the other hand, if it is not IgM clonal, it would be directed either to LC or PC. If there is not any response by 3 months’ time, then switching therapy to the remaining clone would be appropriate [11].

Our patient was treated according to her diagnosis, with a combination of conservative therapy and chemoinmunotherapy. Clone‐directed therapy has demonstrated high hematologic and renal response rates in similar patients with MGRS diagnosis [12], and applying this to our patient, there was no identifiable plasma or B cell clone in the BM examination and she did not have an IgM deposit in the kidneys, which would suggest that her clone was more a plasma cell clone than a B cell clone, favoring the use of plasma clone–directed treatment over a B cell clone one, like rituximab. With these factors in consideration, our patient was started on Daratumumab‐CyborD regimen and fortunately evolved favorably.

In summary, this case exemplifies how MGRS/PGNMID can be a diagnostic challenge to physicians, even as they suspect the presence of plasma cell disorders in patients with acute renal failure. This patient’s presentation can serve as a reminder of why MGRS type syndromes are infrequently thought about when going through a patient’s differential diagnosis list for nephrotic syndrome causes and how important it is to have an experienced pathology service who has previously faced these types of diagnosis. Finally, when treated appropriately, usually patients evolve favorably as it was in this case, and the patient has experienced a normal life and general good health after this complicated diagnosis.

## Funding

No funding was received for this manuscript.

## Disclosure

All authors have read and approved the final version of the manuscript Dr. Agatha Larrazábal had full access to all of the data in this study and takes complete responsibility for the integrity of the data and the accuracy of the data analysis.

## Consent

Patient provided written consent for the writing and publishing of this case report.

## Conflicts of Interest

The authors declare no conflicts of interest.

## Data Availability

The data that support the findings of this study are available on request from the corresponding author. The data are not publicly available due to privacy or ethical restrictions.
